# Glomeruloid microvascular proliferation is associated with lack of response to chemotherapy in breast cancer

**DOI:** 10.1038/bjc.2011.203

**Published:** 2011-06-14

**Authors:** L A Akslen, O Straume, S Geisler, T Sørlie, J-T Chi, T Aas, A-L Børresen-Dale, P E Lønning

**Affiliations:** 1 The Gade Institute, Section for Pathology, University of Bergen, Haukeland University Hospital, Bergen N-5021, Norway; 2 Department of Oncology, Haukeland University Hospital, Bergen, Norway; 3 Department of Genetics, University of Oslo, Oslo, Norway; 4 The Institute of Genome Sciences and Policy, Department of Molecular Genetics and Microbiology, Duke University School of Medicine, Durham, NC, USA; 5 Department of Surgery, Haukeland University Hospital, Bergen, Norway; 6 Institute of Medicine, Section of Oncology, University of Bergen, and Department of Oncology, Haukeland University Hospital, Bergen, Norway

**Keywords:** breast cancer, neoadjuvant chemotherapy, angiogenesis, predictive factor

## Abstract

**Background:**

Glomeruloid microvascular proliferation (GMP), a novel histology-based angiogenesis marker, has been associated with decreased survival in several human cancers.

**Methods:**

In this study, we evaluated the ability of GMP to predict clinical response to neoadjuvant chemotherapy in a series of locally advanced breast cancers (*n*=112).

**Results:**

Presence of GMP (21% of the cases) was significantly associated with high-grade tumours and *TP53* mutations in addition to the basal-like and HER2 subtypes of breast cancer as defined by gene expression data. GMP was correlated to a gene expression signature for tumour hypoxia response. The GMP pattern was also significantly associated with lack of treatment response and progressive disease (*P*=0.004).

**Interpretation:**

The findings suggest that GMP might be able to predict the lack of response to neoadjuvant chemotherapy in locally advanced breast cancer. Whether GMP may be an independent predictor compared with other factors including *TP53* mutation status and tumour grade needs confirmation in larger studies.

Angiogenesis is important for the growth and spread of malignant tumours (Carmeliet, 2003). Although vascular density has been correlated to survival in different malignancies (Weidner *et al*, 1991, 1992; Hlatky *et al*, 2002; Uzzan *et al*, 2004), angiogenesis markers have not been associated with response to chemotherapy or anti-angiogenesis treatment (Paulsen *et al*, 1997; Tynninen *et al*, 2002; Jubb *et al*, 2006). Glomeruloid microvascular proliferations (GMP), a novel angiogenesis marker, are focal aggregates of small vessels resembling a renal glomerulus (Pettersson *et al*, 2000; Sundberg *et al*, 2001). In mice, GMP has been induced by local injection of an adenovirus vector directing VEGF-A expression (Sundberg *et al*, 2001). A human parallel appears to be the POEMS syndrome, where increased VEGF-A is associated with glomeruloid haemangiomas (Tsai *et al*, 2001). In humans, GMP is a defining histological feature of glioblastoma multiforme (Wesseling *et al*, 1993; Schiffer *et al*, 1999) and a prognostic factor in several other tumours (Straume *et al*, 2002; Tanaka *et al*, 2003; Foulkes *et al*, 2004). In this study of locally advanced breast cancer, GMP showed a significant association with lack of treatment response and progressive disease following chemotherapy.

## Patients and methods

### Patients

The patients included in this study were all treated in two prospective single-arm studies for locally advanced breast cancer (T3/T4 and/or N2 tumours) at the Department of Oncology, Haukeland University Hospital (Bergen, Norway). Briefly, in the first study (carried out during 1991–1996), each patient (*n*=94; median age 64 yrs) received doxorubicin monotherapy administered at a dose of 14 mg m^−2^ on a weekly basis (Aas *et al*, 1996; Geisler *et al*, 2001). In the second study (carried out during 1993–2000), 35 patients (median age 67 years) received 5-FU 1000 mg m^−2^ on day 1 and 2 with mitomycin 6 mg m^−2^ day 2 at 3-weekly intervals (Geisler *et al*, 2003). Patients were recruited by the same clinical criteria, with no selection, and tissue was obtained by open surgical biopsy. Clinical responses were classified as complete response (CR), partial response (PR), progressive disease (PD), and stable disease (SD) according to the UICC criteria generally applied at the time period these clinical studies were conducted (Hayward *et al*, 1977). Tumour size in general was measured with use of calipers on a 4-weekly (doxorubicin protocol) or 3-weekly basis (5FU/mitomycin protocol). In case a PD was recorded (25% increase in the product of perpendicular diameters), the patient immediately terminated this chemotherapy and was allocated for an alternative treatment option. For internal consistency, we compared tumours with PD with the combined group of SD/PR/CR, similar to what was conducted in our previous reports from the same materials (Geisler *et al*, 2001, 2003).

### Basic variables and immunohistochemistry

Histological type and histological grade (Elston and Ellis, 1991) were recorded, as well as lymph node status. Staining of endothelial cells by Factor-VIII antibody A-0082 (Dako, Copenhagen, Denmark) was performed on paraffin-embedded archival material (Straume *et al*, 2002). Of a total of 129 cases, sufficient tissue for reliable analysis was available in 112 cases; one histological slide was examined from each case with selection of the highest tumour grade in case of heterogeneity. Positivity for GMP was recorded on the initial surgical biopsy by one observer (OS) and defined as the presence of focal glomerulus-like aggregates of closely associated and multilayered Factor-VIII-positive endothelial cells (Pettersson *et al*, 2000; Sundberg *et al*, 2001; Straume *et al*, 2002). Recorded GMPs or ‘glomeruloid bodies’ typically consisted of 15–100 cells. Glomeruloid microvascular proliferation status was reported as absent or present (one GMP was sufficient to define the case as positive; in most cases more GMPs were observed).

### Mutations and gene expression

Mutations in the *TP53* gene (exons 2–11) were analysed using genomic DNA and TTGE (temporal temperature gradient gel electrophoresis) as reported (Aas *et al*, 1996; Geisler *et al*, 2001). Microarray data were available from previous studies in 78 cases (Perou *et al*, 2000; Sorlie *et al*, 2001, 2003), and analysis of a hypoxia-related expression signature was carried out (Chi *et al*, 2006). The hypoxia signature reflects the most differentially expressed genes in epithelial cells after hypoxia *in vitro* (mammary epithelial cells and renal tubular cells).

### Statistical analysis

Associations between different categorical variables were evaluated by Pearson's *χ*^2^-test, Fisher's exact test or McNemar's test. Statistical significance was assessed at the two-sided 5% level. Prediction of disease progression was analysed by logistic regression. The data were analysed using the SPSS version 15.0 statistical software (SPSS Inc., Chicago, IL, USA).

## Results

Glomeruloid microvascular proliferation positivity was present in 24 of 112 primary tumours (21%) (Table 1). In 56 cases with tissue available after chemotherapy, the frequency of GMP+ increased from 16 to 32% (McNemar's test, *P*=0.035). Regarding histological features, 25% of ductal carcinomas were GMP+, compared with 0% of non-ductal tumours (Fisher's exact test, *P*=0.038). Glomeruloid microvascular proliferation positivity was associated with histological grade (frequency of 0, 14 and 40% in grades 1–3, respectively; *P*=0.001) and lymph node status in particular (pN0–1: 12% GMP+, pN2: 38% GMP+ *P*=0.011) (Table 1).

Glomeruloid microvascular proliferation positivity was significantly associated with lack of response to treatment (PD). Of 17 cases with PD, GMP+ was found in eight cases (47%), compared with 16% among the rest (Pearson's *χ*^2^-test, *P*=0.004). Conversely, 35% of GMP+ cases showed progressive disease, compared with 10% among GMP− cases (*P*=0.004). Further, there was a strong association between GMP+ and *TP53* mutation status (*TP53* mutated tumours, 38% GMP+ *wtTP53* tumours, 11% GMP+ *χ*^2^=11.1, *P*=0.001). This association persisted when mutations in the L2/L3 domain were compared with other mutations and wild-type tumours combined (*χ*^2^=8.8, *P*=0.003). However, GMP+ was also observed in *TP53* wild-type cases (*n*=69) and tended to show an association with progressive disease within this subgroup: two GMP positive of six in the PD group (33%) *vs* five GMP positive of 63 (8%) among the others; Pearson's *χ*^2^-test: *P*=0.049; Fisher's exact test: *P*=0.11. Logistic regression analysis revealed *TP53* mutations (wild type and non-L2/L3 *vs* L2/L3 mutations) to predict disease progression independently (*P*=0.005) (Aas *et al*, 1996; Geisler *et al*, 2003), whereas GMP status showed an independent association with disease progression of borderline significance (*P*=0.07). When cases with either *TP53* mutation (L2/L3 type) or GMP+ were combined, there was a highly significant association with disease progression (PD) (*P*=0.001).

When looking at gene expression patterns in relation to GMP status (78 cases available from the doxorubicin-treated series), statistical analysis of microarrays (SAM) between GMP-positive and -negative tumours revealed that 76 genes were significantly downregulated in GMP-positive cases (none were significantly upregulated; false discovery rate 20%), five of these genes more than twofold: *NAT1* (*N*-acetyltransferase 1), *ESR1* (estrogen receptor 1), *TFF3* (trefoil factor 3), *PLAT* (plasminogen activator, tissue), and *HIST2H2BE* (histone cluster 2, *H2BE*). Of these, two genes (*TFF3*, *PLAT*) have been involved in angiogenesis regulation.

Glomeruloid microvascular proliferation was also associated with a hypoxia-related gene expression signature, which was present in 37% of all tumours and more frequent in GMP-positive cases (55%) when compared with GMP-negative tumours (30%) (*P*=0.041). Positivity for the hypoxia signature was significantly associated with basal-like tumours (83% compared with 29% among the others; *P*=0.0003; the signature was positive in only 3% of luminal A cases).

The GMP frequency was increased among the aggressive basal-like and HER2 subgroups of breast cancer based on gene expression data on these cases (GMP frequency: basal-like 33%, HER2 44%, luminal-A 15%, luminal-B 23% *P*=0.040) (Perou *et al*, 2000; Sorlie *et al*, 2001).

## Discussion

Our findings indicate an association between GMP and lack of clinical response to neoadjuvant chemotherapy in a series of locally advanced breast cancers. In previous studies, no relationship between standard microvessel density and treatment response was observed (Paulsen *et al*, 1997; Tynninen *et al*, 2002; Jubb *et al*, 2006). Here, GMP was significantly correlated to the presence of *TP53* mutations, which might be pathogenetically involved in this angiogenic phenotype (Dameron *et al*, 1994; Foulkes *et al*, 2004). Previous studies have implicated *TP53* in angiogenesis regulation through mechanisms involving TSP-1, bFGF-binding protein, HIF-1α and VEGF (Dameron *et al*, 1994; Ravi *et al*, 2000; Zhang *et al*, 2000; Sherif *et al*, 2001; Pore *et al*, 2004). However, GMP was also observed in mutation-negative tumours, indicating that *TP53* could represent one of several possible pathways. As the hypoxia signature was more frequent in GMP-positive tumours, GMP formation might be stimulated by hypoxia-related pathways, like HIF-1α activation and increased VEGF expression. Taken together, our findings suggest that *TP53* mutations and tumour hypoxia may both be related to the pathogenesis of this angiogenic phenotype in human breast cancer.

Interestingly, in a subset of the cases where tissue was available for study after treatment, the frequency of GMP was significantly increased (from 16% among the initial biopsies to 32% post-treatment in paired samples). It is not clear, however, whether this reflects a sampling effect or a selection of more aggressive and treatment resistant tumour components.

In summary, a significant association between GMP and lack of response to neoadjuvant chemotherapy in locally advanced breast cancer is indicated. Future studies are needed to assess the potential predictive value of GMP for targeted anti-angiogenesis treatment in breast cancer and other tumours.

## Figures and Tables

**Table 1:** Associations between GMP status and important tumour characteristics, as well as treatment response

	**GMP present**		**GMP absent**		
**Variables**	**No.**	**%**	**No.**	**%**	***P*-value^a^**
*Histological type*					0.038
Ductal	24	25	73	75	
Others	0	0	15	100	
					
*Histological grade*					0.001
Grade 1	0	0	17	100	
Grade 2	7	13	45	87	
Grade 3	17	40	26	60	
					
*Lymph node status*					0.011
N0	5	15	28	85	
N1	5	12	37	88	
N2	14	38	23	62	
					
*TP53 mutations*					0.001
Absent	8	11	62	89	
Present	16	38	26	62	
					
*Hypoxia signature*					0.041
Negative	10	20	40	80	
Positive	12	41	17	59	
					
*Molecular subtype* b					0.040^c^
Luminal A	5	15	28	85	
Luminal B	3	23	10	77	
HER2	8	44	10	56	
Basal-like	4	33	8	67	
Normal-like	1	50	1	50	
					
*Clinical response* d					0.004
Non-PD	15	16	79	84	
PD	8	47	9	53	

Abbreviations: GMP=glomeruloid microvascular proliferation; PD=progressive disease.

a *P*-value by Pearson's *χ*^2^-test or Fisher's exact test;

b Molecular subtype is based on microarray data (from Perou *et al*, 2000; Sorlie *et al*, 2001, 2003);

c Basal-like and HER2 tumours combined were compared with the rest;

d Non-PD is a combination of complete response, partial response, and stable disease.
